# Attentional Bias to High-Calorie Food in Binge Eaters With High Shape/Weight Concern

**DOI:** 10.3389/fpsyt.2021.606296

**Published:** 2021-03-04

**Authors:** Chai Lee Seo, Jang-Han Lee

**Affiliations:** ^1^Department of Neuropsychiatry, Seoul National University Hospital, Seoul, South Korea; ^2^Department of Psychology, Chung-Ang University, Seoul, South Korea

**Keywords:** binge eating, shape/weight concern, high-calorie food, craving, attentional bias

## Abstract

Individuals with high shape/weight concern (SWC) place disproportionate emphasis on shape and weight in evaluating their self-worth, making them more vulnerable to body-related cues. Binge eaters (BE), who are obsessed with devouring high-calorie foods, would show severe symptomatology, especially when they have clinically high SWC. The present study attempted to elucidate how SWC influences binging based on attentional patterns toward high-calorie food cues. A total of 120 participants were selected and divided into four groups: (1) BE with high SWC, (2) BE with low SWC, (3) healthy controls (HC) with high SWC, and (4) HC with low SWC. BE and SWC status were respectively determined using the Eating Disorder Diagnostic Scale (DSM-5) and the Eating Disorder Examination Questionnaire. All participants completed the same free-viewing task, measuring initial fixation latency and total fixation duration. BE with high SWC showed attentional bias toward high-calorie food cues in terms of significantly faster initial fixation latency and longer total fixation duration, whereas BE with low SWC and the HC groups did not show any differences. The results revealed that SWC level makes unique contributions to BE's initial orienting bias toward and difficulty disengaging from high-calorie food cues. This may indicate that BE with high SWC merely worry about eating high-calorie food in a cognitive way, but not controlling actual binging behavior. The current study of attentional bias elucidated the role of SWC as a potential maintenance factor of being concerned and binging in BE.

## Introduction

Individuals with high shape/weight concern (SWC) are characterized by overvaluing their shape and weight. Although they worry about gaining weight, SWC is not effective for controlling excessive food consumption (1, 2); instead, the high level of appearance-related distress that SWC entails induces more severe binging symptomatology (3–6). SWC leads individuals to simply worrying about pursuing their concerns on shape-/weight-related cues, rather than to restraining or compensating behavior (7–9). Clinical SWC is regarded as a moderator of binge eating, especially by increasing vulnerability to palatable foods (10, 11). Considering the central role of SWC in binging, binge eaters (BE) with high SWC would be immersed in high calorie. BE have reported elevated craving on palatable food cues, resulting in functional impairment and psychological distress (5, 12, 13). Hyper-reactivity to palatable foods makes BE more vulnerable to these foods and continually concerned with their appearance (7, 8). SWC functions as a driving force of engaging in binging when encountering high-calorie food, specifically working as a moderator of craving (10, 11, 14, 15). People with SWC who have high concern on eating high-calorie food are continually driven to binge due to prepossession with palatable foods (16, 17).

Attentional biases in BE are usually related to stimulus engagement followed by quick avoidance (18–21). Adult BE have shown faster orientation to food cues, reflecting early detection of food stimuli; however, results are mixed on their total fixation duration. For example, overweight and obese BE gazed longer at food stimuli in comparison with weight-matched and normal-weight participants in a free-exploration paradigm and anti-saccadic task (22–24). BE showed prolonged gaze duration on both high- and low-calorie food items compared to controls, thus allocating significantly more attention toward food (18, 23, 24). Another study, however, showed non-significant group differences between BE and controls in attentional bias toward highly palatable food images (20). Similarly, BE-overweight and healthy-overweight groups did not differ significantly on stimulus disengagement from food cues (25). As a moderator of binging, SWC plays a role in pushing attention toward craving-inducing stimuli (26–28). Enhanced allocation of attention toward high-calorie food continues during the maintenance stage due to greater craving (29, 30). SWC prompts BE to experience increased craving toward food cues and to deploy more attention when encountering such cues, possibly leading them to gaze longer at palatable foods (5, 12, 13). To better understand binging as an automatized action, it is crucial to investigate attentional bias toward high-calorie foods, which makes BE more vulnerable to subsequent binging episodes (10, 22).

To investigate attentional responses to high-calorie food in BE with different SWC levels, a free-exploration paradigm using eye tracking is an appropriate method (31). Facilitated attention refers to the fast detection of salient cues through automatic processing in the initial stage, whereas disengagement involves strategic processing in the maintained stage of attention. Accordingly, disengagement difficulty refers to continuous attention on disorder-salient cues. BE with high SWC are expected to initially orient toward high-calorie food cues in the initial stage and then maintain their attention. The present study investigates the role of SWC on binge eating behavior by focusing on the attentional bias, especially toward high-calorie food. The research hypotheses are as follows.

*H*_1_*:* BE with high SWC orient their attention to high-calorie food cues faster than BE with low SWC.
*H*_2_*:* BE with high SWC have greater difficulty disengaging from high-calorie food cues and so maintain attention on them.

Previous studies have used time intervals to investigate information processing with specific initial and late stages; however, the current study adopts total fixation duration for elucidating prolonged gaze during the allowed time. For this purpose, the present study used an attentional free-viewing paradigm with an eye tracker, which is useful to distinguish attentional patterns over a total time period (31).

## Methods

### Participants

Prior to the experiment and as an initial screening measure for BE with high and low SWC, a total of 716 female adults completed the Eating Disorder Diagnostic Scale (DSM-5, EDDS; 32) and the Eating Disorder Examination Questionnaire (EDE-Q; 33). The inclusion criterion for BE was to have binge eating episodes with three or more symptoms at least once a week for 3 months (32). The presence of SWC was assessed using the EDE-Q SWC subscales. Scores above 30 (highest 30%) on the EDE indicate clinically significant levels of overconcern with shape and weight; scores below 19 (lowest 30%) indicate comparatively low levels of concern with shape and weight. Based on this sampling method, the following four groups were formed: (a) BE with high SWC, (b) BE with low SWC, (c) healthy controls (HC) with high SWC, and (d) HC with low SWC. A total of 120 participants completed the experiment. Exclusion criteria in the present study were as follows: (1) diagnosis of other eating disorders (EDs), (2) recurrent use of inappropriate compensatory behavior, and (3) receipt of any pharmacological treatment. All participants gave informed consent to participate in the experiment. The study was approved by the Institutional Review Board of Chung-Ang University, Seoul, Republic of Korea (no. 1041078-201910-HRSB-320-01).

### Questionnaires

#### EDDS

The EDDS is a 22-item self-report measure used to assess sub-threshold and full-threshold diagnostic classifications of anorexia nervosa (AN), bulimia nervosa (BN), binge eating disorder (BED), and any other EDs (32). The Korean version of the EDDS (K-EDDS) was used in this study (33). Questions were answered using Likert-type scales, yes or no responses, or frequency ratings. Overall symptoms were calculated from the sum of scores for the first 18 items. In the current research, only individuals classified with BE on the EDDS were included. That is, individuals with any other ED symptoms such as recurrent use of inappropriate compensatory behaviors—at least once a week for 3 months—were excluded. Cronbach's α was 0.84.

#### EDE-Q Self-Report Version

The EDE-Q is a 36-item measure derived from the EDE interview and focuses on the past 28 days (34). The Korean version of the EDE-Q version 6.0 was used in the current study (35). Items addressing eating disorder attitudes are scored on a 7-point Likert scale, forced choice, and ratings. Subscale and global scores were derived in scores of 4 or higher on key items, indicating clinical severity. Frequency for eating-related behaviors was considered as the number of episodes during the past 4 weeks. The presence of SWC was assessed by the scores of EDE-Q SWC subscales. Cronbach's α was 0.91.

#### Hunger Visual Analog Scale (VAS)

Participants were asked to give ratings in response to four questions: “How hungry are you?” “How satiated do you feel?” “How full do you feel?” and “How much do you want to eat?” Ratings were given on a VAS that ranged from 0 mm (“not at all”) to 100 mm (“very much”) (36). The scale evaluated the difference in hunger between the BE groups and the HC groups.

#### Beck Depression Inventory (BDI)

The BDI is a 21-item questionnaire that was originally developed to assess the presence and severity of depression symptoms in a clinical population (37). The Korean version of the BDI was used in the present study (38). The scale assessed the cognitive, emotional, and somatic symptoms of depression. Each item had four choices that describe the severity of the respective symptoms. Participants chose one option that best described their personal state during the past week. Cronbach's α was 0.86.

#### State–Trait Anxiety Inventory (STAI-X-1 and STAI-S)

The STAI-S was measured to rule out participants' situational state of anxiety (39). The Korean version of STAI was used (40). The STAI-S included 20 items rated on a 4-point Likert scale ranging from 1 (not at all) to 4 (very much). The total scores of the scale ranged from 20 to 80. Higher scores indicated more severe anxiety. Cronbach's α was 0.82.

#### General Food Cravings Questionnaire-Trait (G-FCQ-T)

Food craving was assessed with the Korean version (41) of the General Food Cravings Questionnaire-Trait [G-FCQ-T; (42)]. The measure comprised 39 statements assessing the intensity of desire for food over time and in various situations, considering trait-related behavior. Items were scored on a 6-point scale from 1 (“never/not applicable”) to 6 (“always”). Higher scores indicated more frequent and intense food cravings. Cronbach's α was 0.89.

#### Body Mass Index (BMI)

BMI was assessed in order to match weight between groups (43). BMI was calculated using participants' self-reported weight and height. The formula is *BMI* = *weight (kg)/height (m) squared*. Self-reported height and weight are extremely reliable and accurate, and very few people under-report weight by more than 10% (44).

### Eye-Tracking Task

#### Free-Viewing Task

**Figure 1:**
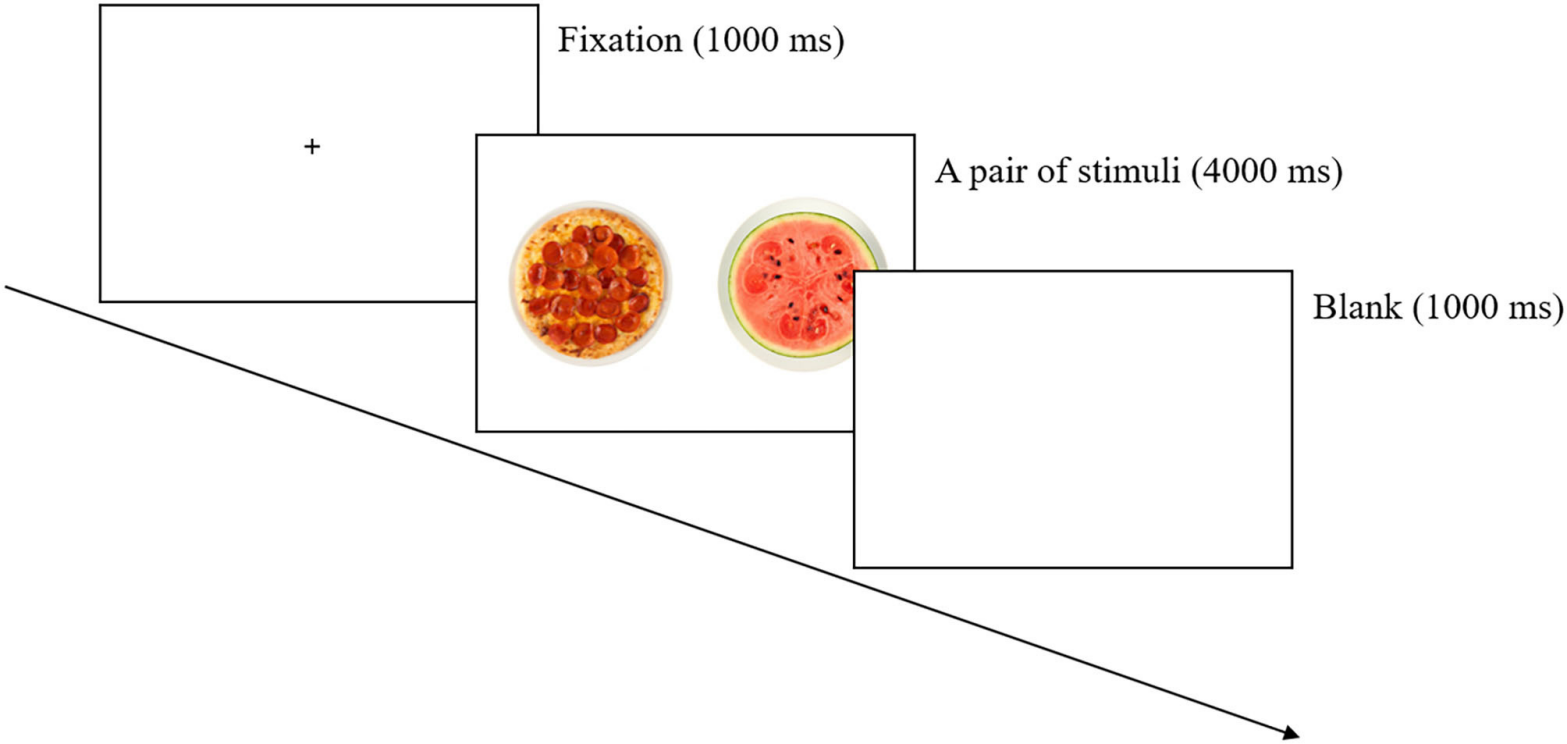
Procedure for the free-viewing task.

For the free-viewing task, all stimulus images were selected from the *FATIS* addictive image database (45). The *FATIS* is a database of pictures with normed ratings on addictive images including food, alcohol, and nicotine and non-addictive neutral items. High- and low-calorie cues were determined based on the actual and perceived calories of specific foods, as rated and standardized in the *FATIS*. Pairs of high- and low-calorie food cues and neutral images were matched as closely as possible for color, complexity, brightness, and size (see Supplementary Material 1). Each pair was presented in a counterbalanced order, and cues were presented twice on each side of the monitor. Each pair of cues was presented at a size of 80 × 100 mm with their centers 200 mm apart. A total of 27 images were used as stimuli: nine high-calorie food images, nine low-calorie food images, and nine neutral images such as households. The high-calorie food cues were all items containing high quantities of fat and sugar. The low-calorie food cues included various types of vegetables and fruits. The matched pairs between high- and low-calorie food images are as follows: (1) pizza–watermelon, (2) ice creams–boiled eggs, (3) chocolate bars–dotorimuk (acorn jelly), (4) sausages–carrots, (5) cream pasta–bean sprouts, (6) chocolate truffles–walnuts, (7) potato wedges–bananas, (8) cream puffs–mushrooms, and (9) cookies–grapes (see Supplementary Material 1). The free-viewing task was used to record the participant's natural eye movement in response to food and neutral pictures over time. A fixation cross (+) appeared in the center of the screen for 500 ms. After the fixation period, a pair of images was presented for 4,000 ms, followed by a blank screen for 1,000 ms. Participants were asked to look freely at the pictures on the screen (see Figure 1). A total of 54 trials were conducted, following Kim et al. (46).

During the free-viewing task, initial fixation latency and total fixation duration toward each stimulus were collected. Initial fixation latency has been used as an initial orientation measure in terms of automatic and unconscious approach tendency (15, 36, 47, 48). Although people cannot anticipate the position of each food picture, they automatically fixate their attention toward a more self-vigilant cue when the pairs come up. Total fixation duration is a measure of sustained attention, a more conscious and motivated attention compared to the initial fixation latency (49, 50).

#### Apparatus

During the task, participants' eye movements were recorded by an eye-tracking system (Tobii TX300, Tobii Technology AB, Danderyd, Sweden). This integrates the infrared sensors, the camera, and the eye tracker, which allows participants to naturally move their heads and eyes without any attached sensors. Participants' eyes were ~65 cm from the monitor, which was 23 inches in size (1,920 × 1,080 pixels). Eye tracking was measured at 120 Hz. The criterion for fixation was a stable gaze lasting at least 80 ms, at an angle of 1.4° (29). Before the task started, the eye-tracking equipment was calibrated for each participant by presenting five dots on the screen. Data were processed using Tobii Studio (Tobii Technology AB, Danderyd, Sweden). In the current study, gaze duration was defined as the sum of durations from all fixations that hit the area of interest (49). Areas of interest (AOIs) were designated for each trial and corresponded with the location of each food cue (i.e., the size of either high- or low-calorie food cues).

### Procedure

Participants were asked to fast for 6 h prior to participating in the experiment, in order to control their satiety level. When participants arrived at the laboratory, they completed an informed consent form, approved by the institutional review board of Chung-Ang University, before participation. They were also asked to fill out self-report questionnaires on hunger (VAS), current anxiety level (STAI-S), and recent depression (BDI). Each participant was then asked to sit at a desk and position their face ~60 cm away from the computer monitor. The eye-tracking system was calibrated for recording. Here, participants were asked to look at the monitor freely, adopting a fixed posture to minimize movement during the free-viewing task. After completing the task, participants were fully debriefed about the experiment. The experimental procedure took ~40 min, and all participants were given 10,000 KRW (~USD 10) as a monetary reward.

### Data Analysis

The required sample size for the present study was calculated using G^*^Power 3.1.9.4 (University of Dusseldorf, Dusseldorf, Germany), with an alpha error probability of 0.05 and power of 0.95. A large effect size ($\eta_{\text{p}}^{\;2}$ = 0.40) was expected with the current sample size. A 2 (group: BE and HC) × 2 (SWC: high and low) mixed analysis of variance (ANOVA) on high-calorie food cues was utilized to analyze the data. To investigate specific attentional bias toward high-calorie food cues, the main analysis was conducted only for high-calorie stimuli, while the other cues were used as dummy variables (see Supplementary Materials 2, 3 for low-calorie and neutral stimulus data, respectively). In the free-viewing task, fixation latency and total fixation duration on the AOIs were analyzed (i.e., the locations of high- or low-calorie food cues or neutral cues). In total, 54 trials were analyzed.

First, independent *t*-tests were conducted to examine differences between the BE and HC groups in terms of age, BMI, BDI, and STAI-S. Next, eye-movement data in the free-viewing task were analyzed. Initial fixation and total fixation duration on high-calorie food cues are an indicator of current motivation to pursue rewarding experiences in response to appetitive cues, such as approach tendencies (15, 36, 48). Moreover, it is necessary to differentiate between automatic and deliberate responses according to the temporal properties of visual attentional processes in order to establish whether BE with high SWC exhibited increased attention toward high-calorie foods automatically and not deliberately, whereas BE with low SWC maintained the approach toward high-calorie food. Initial fixation duration is calculated as the total time of all saccades and very first fixations (47). The total fixation duration is calculated as the sum duration of all fixations on AOIs (49). Additionally, any significant interaction effects were examined using *post-hoc* Bonferroni analyses, and degrees of freedom were adjusted with the Greenhouse–Geisser epsilon to correct for violations of the sphericity assumption. All analyses were performed in IBM SPSS version 25.0.

The analyses discarded 15 participants: five were diagnosed with other psychiatric disorders, four were taking medications related to appetite, two had missing data due to task error, and four had a gaze duration percentage >2 standard deviations from the mean. The statistical analyses were conducted for 105 participants: 25 BE with high SWC, 25 BE with low SWC, 25 HC with high SWC, and 30 HC with low SWC.

## Results

### Demographic and Group Characteristics

The demographic characteristics of participants were analyzed by age, BMI score, current hunger level, depression, state anxiety, and trait food craving (see Table 1). The results indicated that there were no significant differences among the four groups in mean age, BMI score, current hunger level, and depression level. Although there was a significant difference in state anxiety among the groups [*F*_(3, 101)_ = 2.836, *p* < 0.05, $\eta_{\text{p}}^{\;2}$ = 0.074], this disappeared in *post-hoc* analysis. The results suggest that the findings of the current study are not explained by these demographic and other psychological factors. There was a significant statistical difference in trait food craving [*F*_(3, 101)_ = 18.558, *p* < 0.001, $\eta_{\text{p}}^{\;2}$ = 0.365]. In *post-hoc* analysis, BE with high SWC showed a higher food craving level compared to the other groups. Both BE groups had a statistically higher craving level than both HC groups, while HC with high SWC reported higher food craving than did HC with low SWC. Both binge eating behavior and high SWC were evidently related to trait food craving.

**Table 1:** Demographics and clinical characteristics for each group.

	**BE**		**HC**		***F***	***post-hoc***
	**1** **High SWC (*n* = 25)**	**2** **Low SWC (*n* = 25)**	**3** **High SWC (*n* = 25)**	**4** **Low SWC (*n* = 30)**		
Age	21.13 (2.06)	21.68 (2.14)	21.81 (2.28)	21.73 (2.31)	0.51	
BMI	20.79 (1.78)	22.06 (2.45)	20.80 (2.12)	21.53 (2.65)	1.97	
Hunger	75.26 (13.40)	75.83 (11.75)	77.01 (11.79)	73.65 (13.54)	0.34	
BDI	12.23 (6.62)	10.60 (5.92)	9.04 (8.76)	9.10 (6.22)	1.38	
STAI-S	45.53 (9.49)	42.48 (5.96)	40.54 (6.60)	40.50 (7.51)	2.84	
Food Craving	79.26 (17.07)	79.48 (14.39)	63.04 (13.87)	55.73 (11.29)	18.56^***^	1, 2 > 3, 4
EDDS	6.50 (1.14)	6.28 (1.02)	2.31 (1.72)	2.83 (1.78)	63.67^***^	1, 2 > 3, 4
EDE-Q	32.53 (2.81)	13.04 (5.83)	30.31 (7.94)	15.50 (4.02)	95.62^***^	1, 3 > 2, 4

Mean (standard deviation);

*** *p < 0.001*,

** *p < 0.01*,

* *p < 0.05. BE = binge eaters; HC = healthy controls; SWC = shape/weight concern; BMI = body mass index; Hunger = Hunger VAS; BDI = Beck Depression Index; STAI-S = State–Trait Anxiety Inventory-State; Food Craving = General Food Cravings Questionnaire-Trait; EDDS = Eating Disorder Diagnostic Scale-Binging Behavior Subscale; EDE-Q = Eating Disorder Examination Questionnaire-Shape/Weight Concern Subscale. Test Statistics (F) = results of the omnibus F-test*.

In the analysis of clinical measures, the results for binging behavior differed significantly between the BE and HC groups. Specifically, the BE groups had higher binging behavior [*t*_(103)_ = 13.737, *p* < 0.001, Cohen's *d* = 2.632] than the HC groups. Also, the two high-SWC groups showed higher SWC [*t*_(103)_ = 4.709, *p* < 0.001, Cohen's *d* = 0.915] than the two low-SWC groups. These results confirm that the sampling method successfully allocated participants to the four groups according to their clinical status.

### Initial Fixation Latency

**Figure 2:**
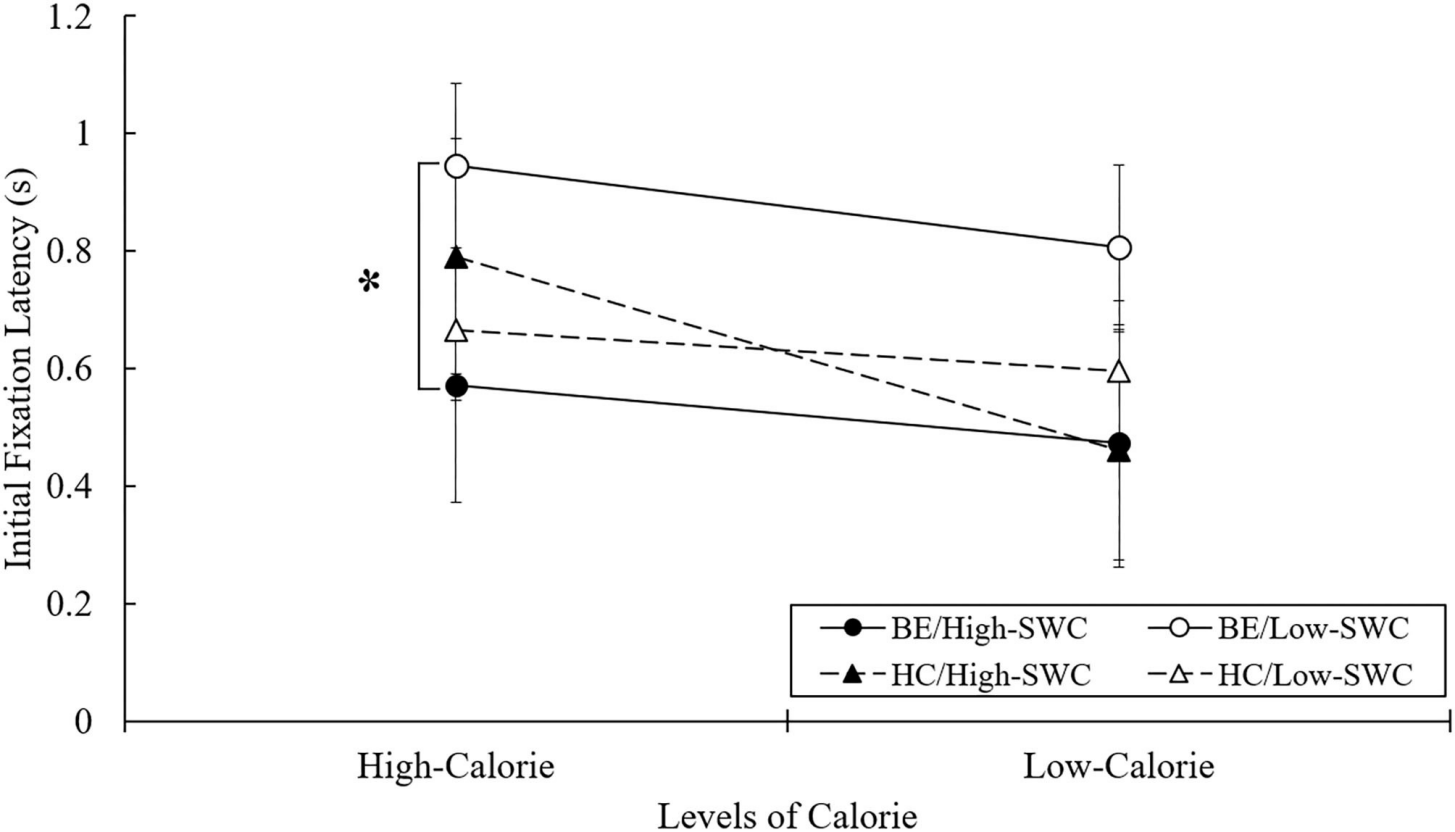
Mean initial fixation latency in free-viewing task. BE, binge eaters; HC, healthy controls; SWC, shape/weight concern. *p < 0.05.

Initial fixation latency was the time interval that participants spent locating their initial fixation toward each stimulus. Two-way ANOVAs revealed an interaction effect between BE and SWC levels [*F*_(3, 101)_ = 6.679, *p* < 0.05, $\eta_{\text{p}}^{\;2}$ = 0.062] for high-calorie stimuli. In *post-hoc* analysis, the initial fixation latency of BE with high SWC was lower than that for BE with low SWC (*p* < 0.05), showing that initial fixation latency toward high-calorie food cues was shorter for BE with high SWC. By contrast, the scores between the two HC groups did not statistically differ (see Figure 2). These results showed that the SWC level made a unique contribution to the initial orienting bias toward high-calorie food cues only in BE.

### Total Fixation Duration

**Figure 3:**
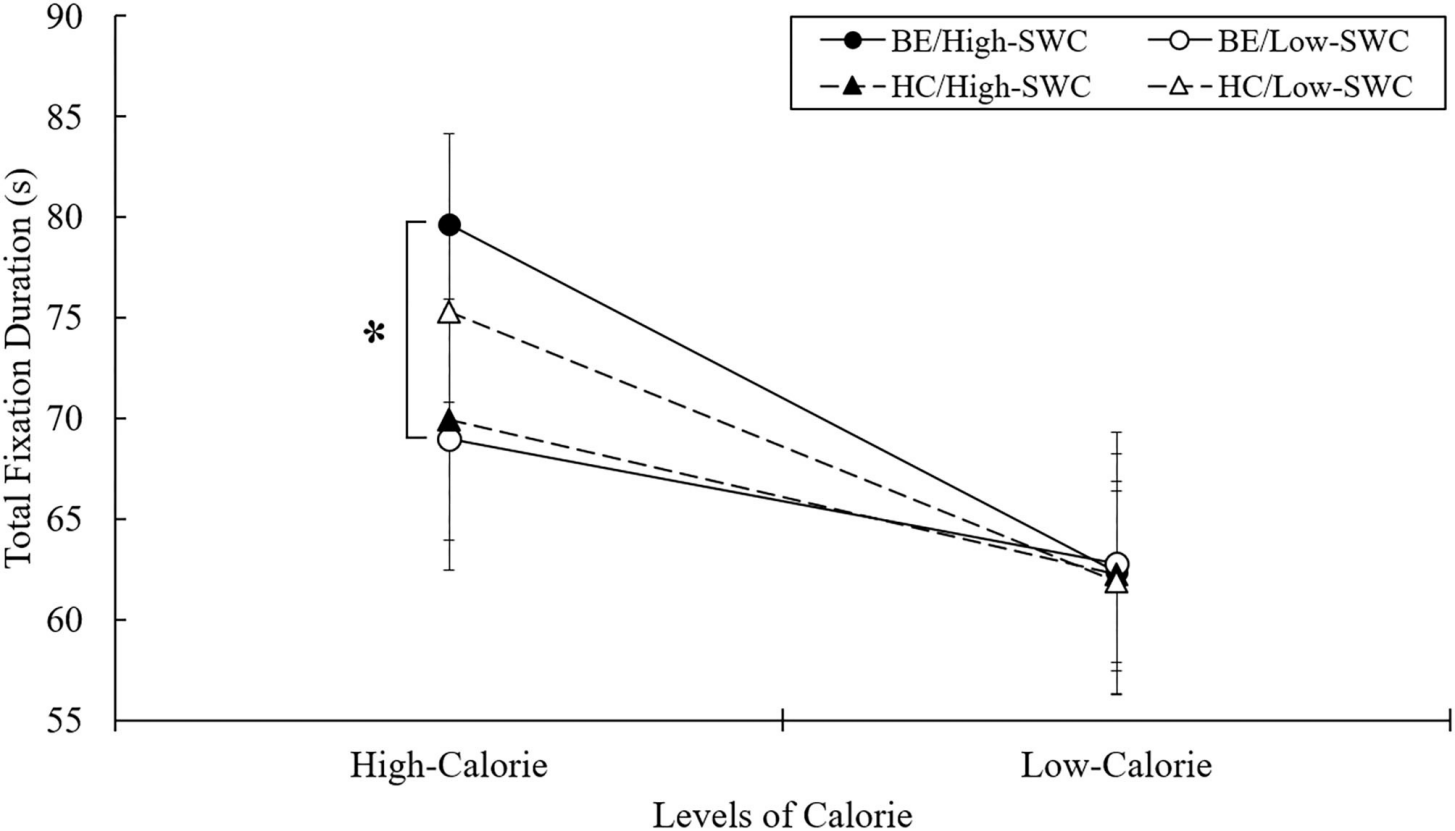
Mean total fixation duration in free-viewing task. BE, binge eaters; HC, healthy controls; SWC, shape/weight concern. *p < 0.05.

Total fixation duration was the sum duration of all fixations on each stimulus. This allowed for the assessment of gaze duration for each pair of picture types over 4 s. Two-way ANOVA analyses revealed an interaction effect between BE and SWC levels [*F*_(3, 101)_ = 5.787, *p* < 0.05, $\eta_{\text{p}}^{\;2}$ = 0.054] for high-calorie stimuli. The total duration time of gazing at high-calorie food cues was significantly longer for BE with high SWC than for the BE with low SWC, whereas the two HC groups did not differ in this regard (see Figure 3). BE with high SWC evidently found it most difficult to disengage from high-calorie food cues, whereas BE with low SWC were able to disengage from high-calorie food cues just as HC groups were.

## Discussion

The present study was designed to examine whether attentional bias for high-calorie food cues was affected by SWC in BE. The results showed that BE with high SWC exhibited attentional bias toward high-calorie food cues in terms of both initial fixation latency and total fixation time. BE with high SWC showed shorter initial fixation latency and longer total fixation time toward high-calorie food cues, whereas BE with low SWC and the two HC groups (with high and low SWC) did not differ significantly.

Due to constant attention toward the cues, BE with high SWC showed difficulty disengaging from high-calorie food (22, 28, 30). Craving is closely related to giving undue importance to shape and weight. Persistent cognitive concern over shape/weight is induced by increased craving, while craving is closely related to overvaluation of shape and weight (2). BE with high SWC rather maintained engagement over time and cognitively pursued high-calorie food cues. Even though they put a great amount of value on shape and weight, they did not intentionally inhibit approach responses, but merely concerning their appearance (51, 52). They seemed to be more likely to maintain attention on the powerful reward cues of high-calorie food. The present study suggests repeated and constant attention in high-calorie food as the potential underlying mechanism for recurrent binging episodes.

BE with high SWC showed shorter initial fixation latency toward high-calorie food cues than BE with low SWC. Importantly, the strengthened approach toward high-calorie food in BE with high SWC may result from vigilant attention toward palatable foods (16, 22). Furthermore, BE with high SWC fixated on high-calorie food cues for significantly longer in total than BE with low SWC. Impaired disengagement may be a critical vulnerability for individuals at risk of binging, in light of evidence that attentional bias toward high-calorie food cues contributes to the development and maintenance of body dissatisfaction and binge disorders (53).

Interestingly, BE with high SWC showed unique attentional patterns. All EDs tend to be associated with a spontaneous and non-volitional response to food-related cues; however, EDs accompanied by restrained eating or compensatory behavior tend to result in avoidance of high-calorie food that manifests in the strategic stage of attention (22, 46, 54). Distress intolerance may encourage BED as a way to cope with experiencing intense states of distress and severe instability, whereas clinical perfectionism and fear of negative evaluation are the dominant factors of maintaining AN and BN (55, 56). When BE with high SWC encounter high-calorie food cues, continuous attention toward shape-/weight-related cues induces greater biological craving. Since inhibition of craving causes distress to BE with high SWC, they immediately and persistently approach toward high-calorie food to quickly reduce the discomfort, instead of enduring their dysfunctional craving. By contrast, individuals with AN or BN are more likely to be vigilant to external evaluation and pursue perfectionism. Due to underlying factors, they employ a strategy of dietary restraint or compensatory behavior, thus activating the inhibitory system (55–57). Combining the present study's results with prior findings, it appears that AN and BN are relatively homogeneous subtypes whereas BED is a distinct disorder (57). In sum, even though SWC is a common characteristic among individuals with EDs, BE with high SWC may potentially develop and maintain binging behavior due to attentional bias toward palatable foods and continuous craving.

Attentional bias in the orientation stage and maintenance stage is regarded as automatic/unconscious and strategic/conscious attention, respectively. However, the present study did not measure participants' information processing with early and late stages toward high-calorie food and so could not determine whether they have different attentional patterns over each time interval. To advance beyond the current study's use of total fixation duration for elucidating prolonged gaze, future studies should investigate BE's attention toward food cues during different time intervals.

There are several limitations to the present study. First, it did not consider actual food intake among BE with different SWC levels. Attentional bias toward high-calorie foods is thought to be a factor in the development and maintenance of binging behavior, but the current study did not elucidate whether SWC will actually affect binging of palatable foods. Future studies could use a bogus taste test to provide additional evidence of how SWC influences binging behavior. Second, the power of the eye-tracking task was somewhat limited. It only included 27 pictures in total with 54 trials, which are deemed few for psychophysiology. The number of trials was decided considering participants' boredom and fatigue during the task (46). The future study could include more pairs of cues and eye-tracking trials. Third, the BE group still showed mild to moderate depressive symptoms. The influence of other psychiatric illnesses might not be removed from the BE groups' attentional bias. In a further study, the depression and anxiety symptoms should be controlled thoroughly in order to clarify the result. Fourth, although the present study attempted to control participants' current hunger level by asking them to fast for at least 6 h before the experiment, time since the last food intake varied among participants: some of them fasted for 6 h whereas others fasted much more than 6 h. Although participants' satiety levels did not significantly differ, future studies should control participants' satiety levels using the last food intake time. Fifth, since the central cross was not set as an area of interest, the initial fixation latency to the central cross was not provided. It would offer bountiful results and interpretation whether the study clarifies that larger fixation latencies to food images can be observed to neutral orienting stimuli. Sixth, the measure of initial fixation latency may be insufficient for representing attentional orientation. When unaware of each stimulus, participants would turn their attention based on physical properties of the stimulus which can be processed more early and rapidly. It is necessary to ensure that initial orientation occurred by an automatic seeking tendency toward a more self-vigilant cue in a further study. Finally, images in the current study were used as matched pairs in terms of color, complexity, brightness, and size; the stimuli were not rated by the participants. The arousal level, valence level, and any other physical properties could affect the result. Future studies should include participants rating stage in the experiment in order to control those effects.

To conclude, the current study's findings suggest that high-calorie food perception is biased in female BE compared to weight-matched controls of the same gender. SWC level makes unique contributions to the initial orienting bias toward and difficulty disengaging from high-calorie food cues in BE. This may indicate that BE with high SWC merely retain their concern about high-calorie food rather than control their persistent attention toward these cues. This is the first empirical evidence on SWC and differences in attentional patterns between BE and weight-matched HCs. The results elucidate the role of SWC as a maintenance factor of being concerned and binging in BE. As visual food cues are particularly prominent in modern society, understanding the cognitive process of BE with high SWC when exposed to such cues is of great importance in developing potential behavioral therapies, environmental alterations, and public health measures. Based on the results, attention bias modification and cognitive schema modification could also be implemented to modify the specific attentional bias toward high-calorie food and subsequent binging behavior.

## Data Availability Statement

The raw data supporting the conclusions of this article will be made available on request to the corresponding author.

## Ethics Statement

The studies involving human participants were reviewed and approved by Institutional Review Board of Chung-Ang University. The patients/participants provided their written informed consent to participate in this study.

## Author Contributions

CS and J-HL: conceiving the experiment and data interpretation and drafting the manuscript. CS: design of the experimental task, data collection, and analysis of data. Both authors revised the manuscript critically and gave final approval of the version to be published.

## Conflict of Interest

The authors declare that the research was conducted in the absence of any commercial or financial relationships that could be construed as a potential conflict of interest.

## Supplementary Material

Supplementary Material 1 Matched pairs of high- and low-calorie food cues and neutral items.

Supplementary Material 2 Mean initial fixation latency for each stimulus among groups.

Supplementary Material 3 Mean total fixation duration for each stimulus among groups.
