# Supplementary material for: Attentional Bias to High-Calorie Food in Binge Eaters With High Shape/Weight Concern
Source: Front Psychiatry. 2021 Mar 4;12:606296. doi: 10.3389/fpsyt.2021.606296 (PMC7982957; doi:10.3389/fpsyt.2021.606296)
Supplement: Supplementary Material 1 — Matched pairs of high- and low-calorie food cues and neutral items. [file Table_1.DOCX]

Supplementary 1 | Matched pairs of high- and low-calorie food cues and neutral items.

| **High-calorie vs. Low-calorie** | **High-calorie vs. Neutral** | **Low-calorie vs. Neutral** |
| --- | --- | --- |
| 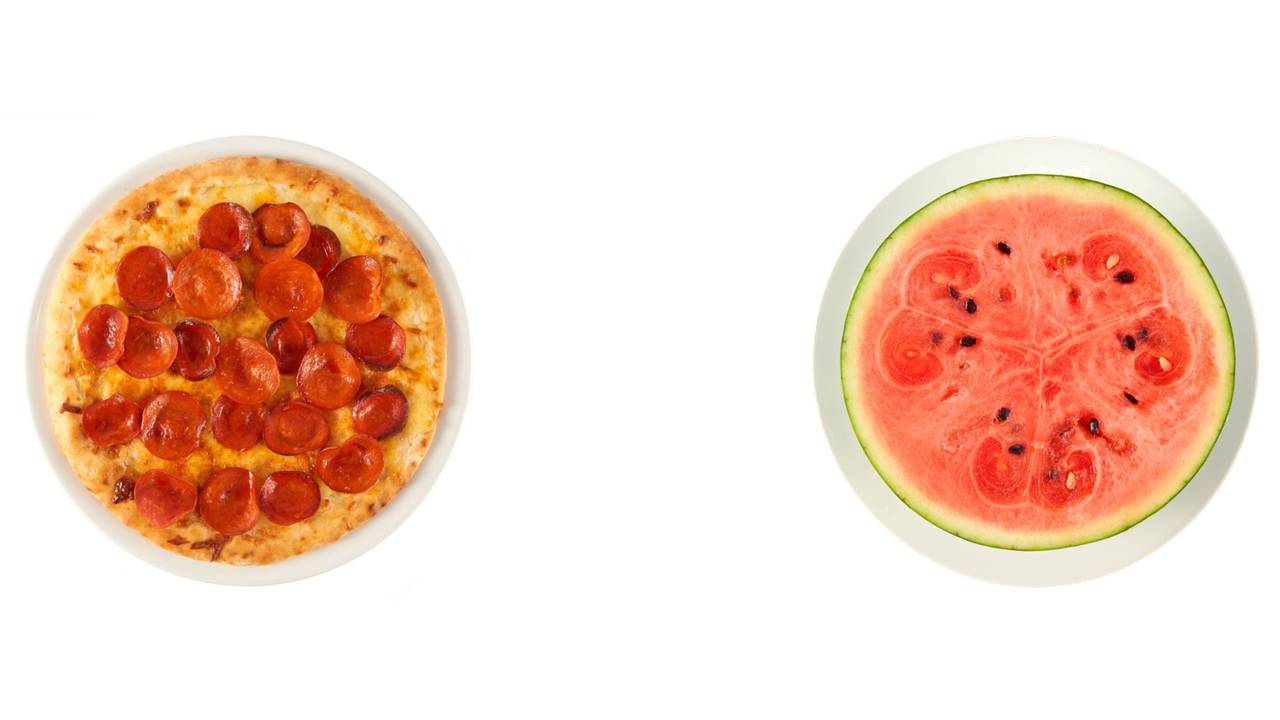  Pizza - Watermelon | 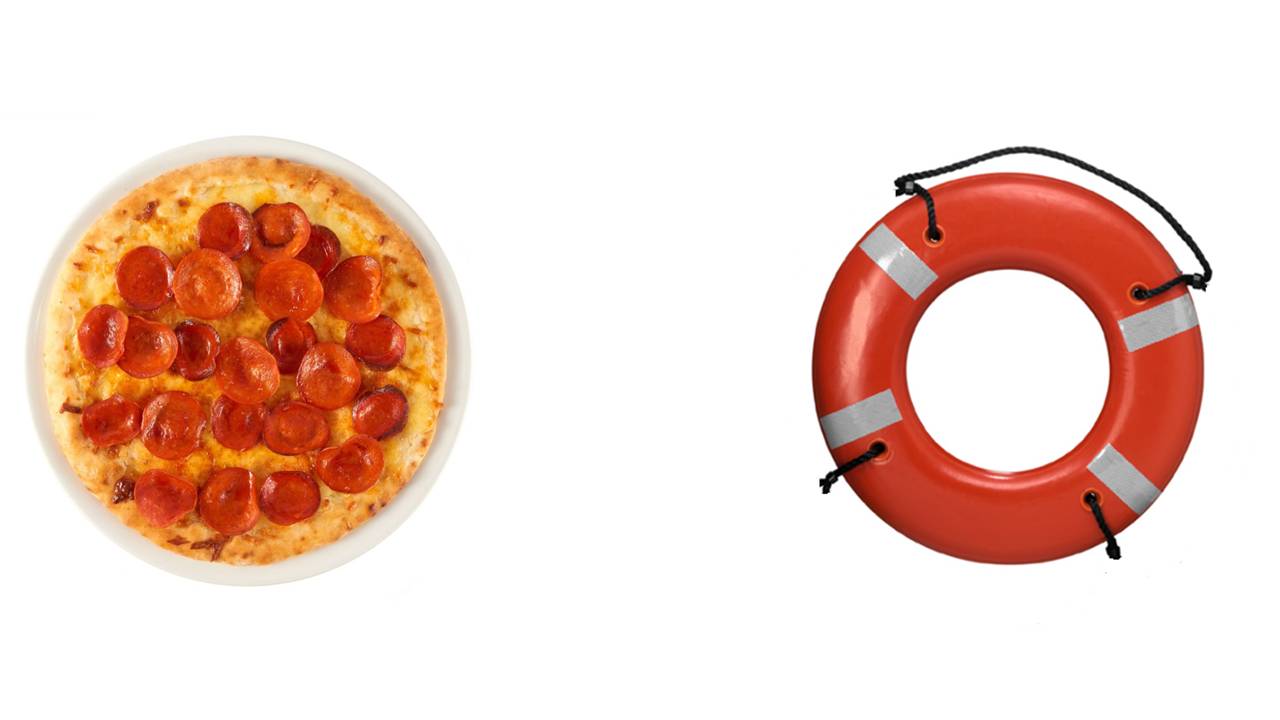  Pizza – Life ring | 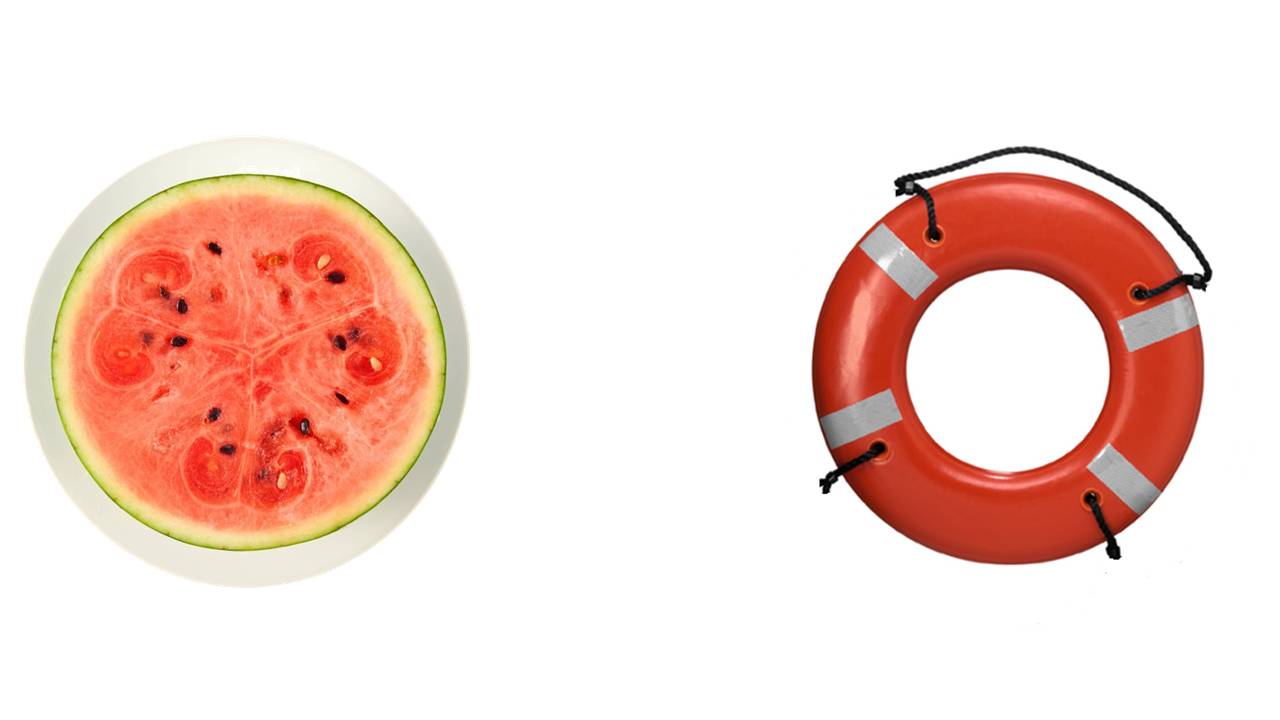  Watermelon – Life ring |
| Ice creams 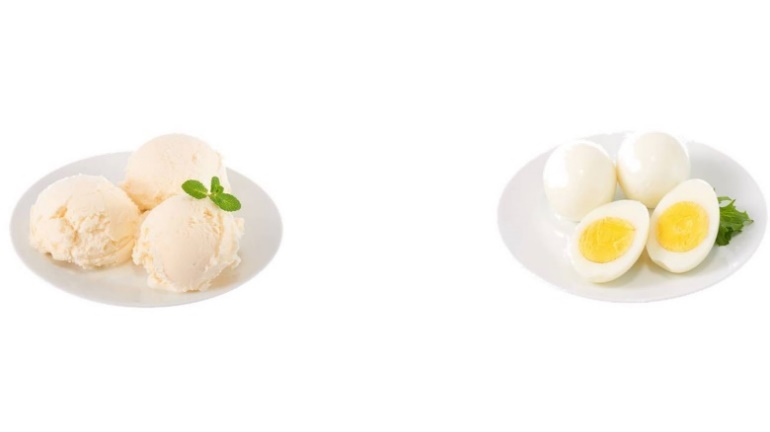– Boiled eggs | 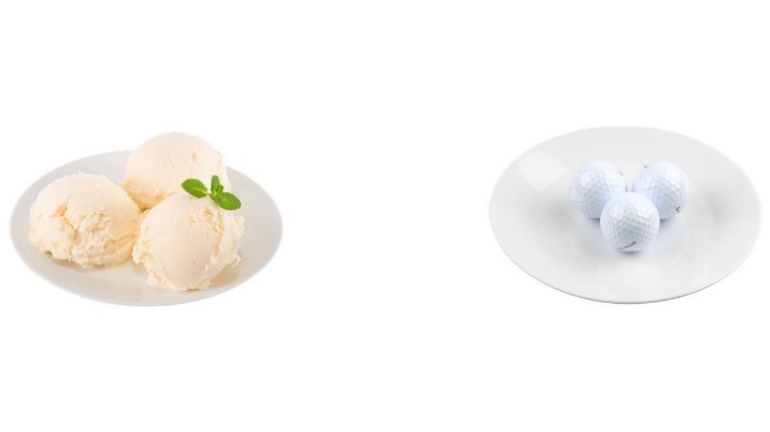  Ice creams – Golf balls | 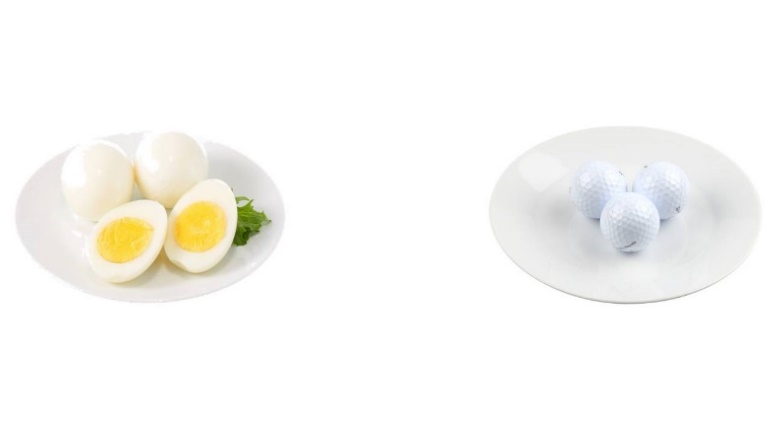  Boiled eggs – Golf balls |
| 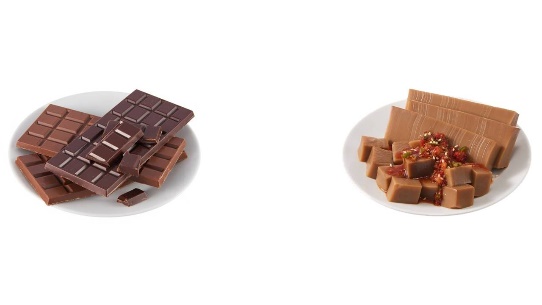  Chocolate bars - Dotorimuk | 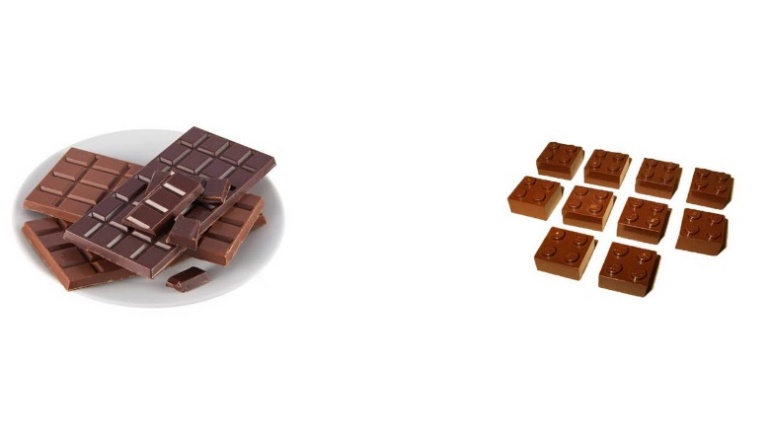  Chocolate bars – Toy bricks | 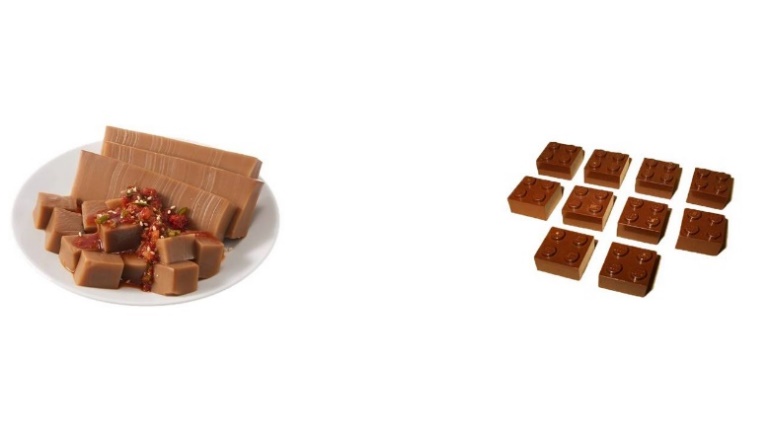  Dotorimuk – Toy bricks |
| 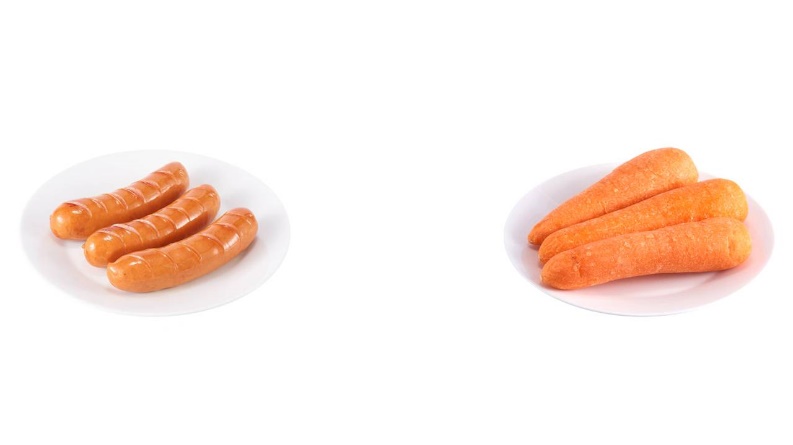  Sausages - Carrots | 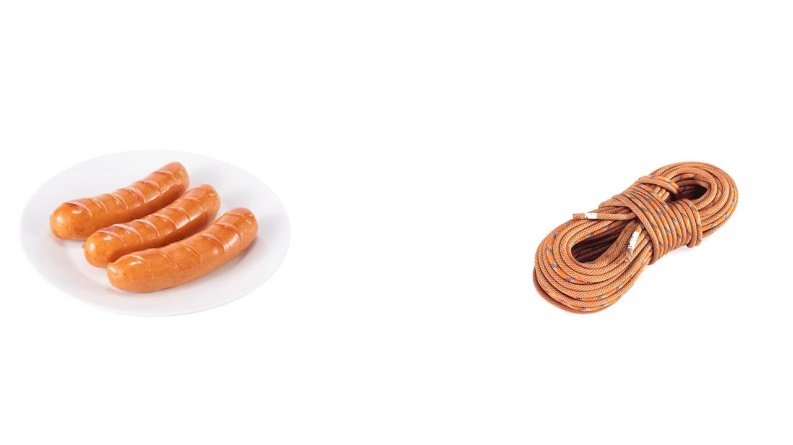  Sausages - Ropes | 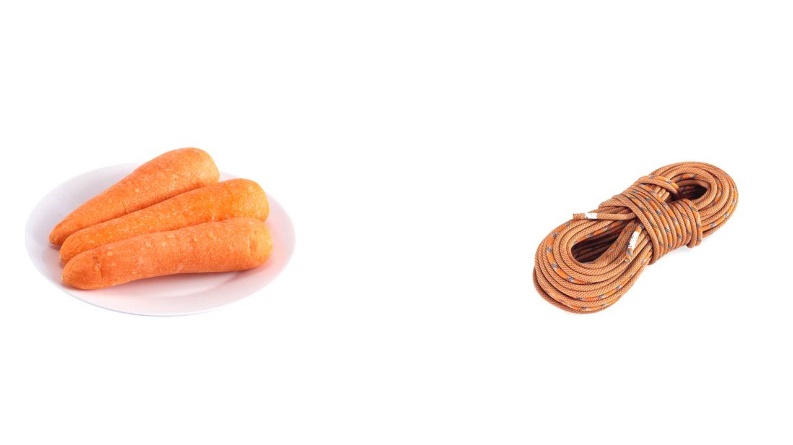  Carrots - Ropes |
| 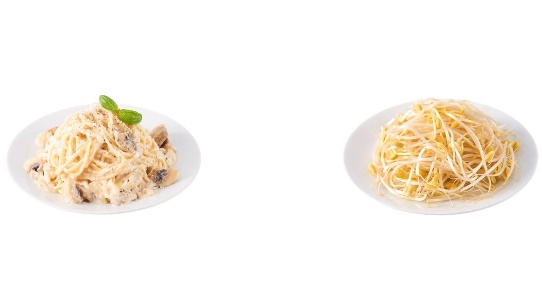  Cream pasta – Bean sprouts | 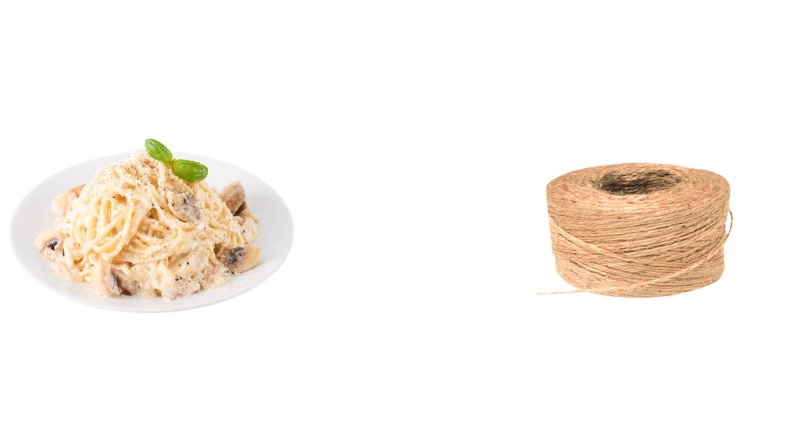  Cream pasta – Packthread | 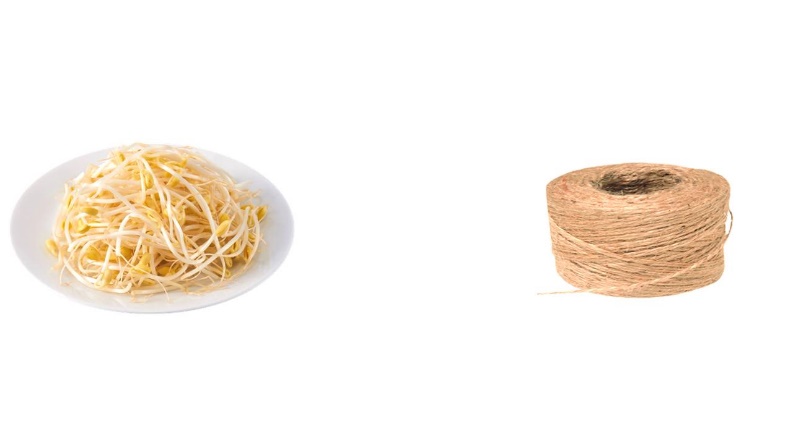  Bean sprouts - Packthread |
| 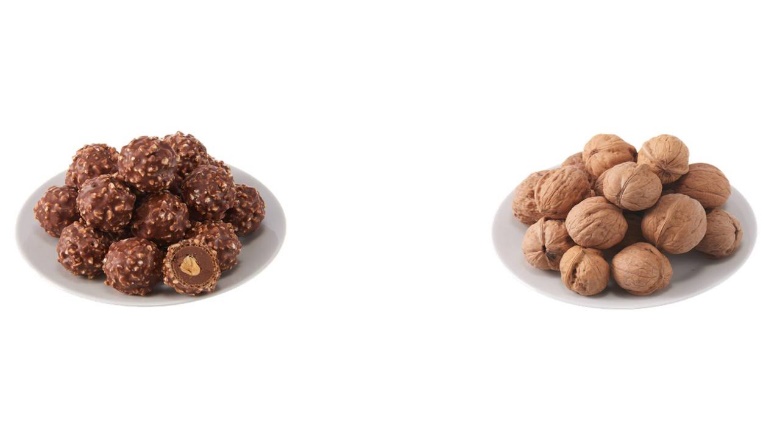  Chocolate truffles - Walnuts | 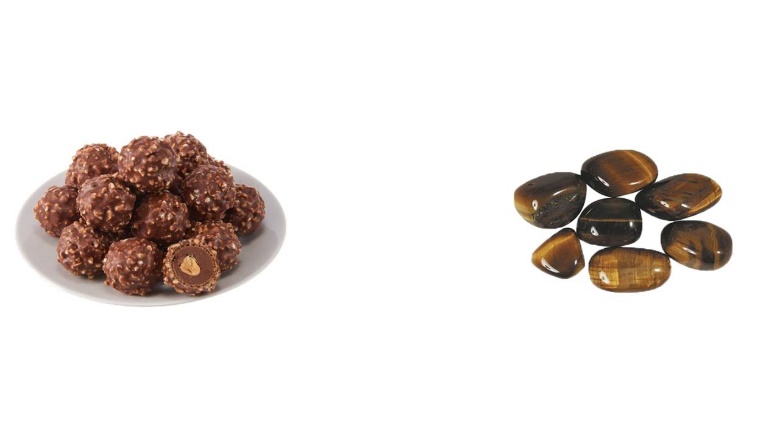  Chocolate truffles - Stones | 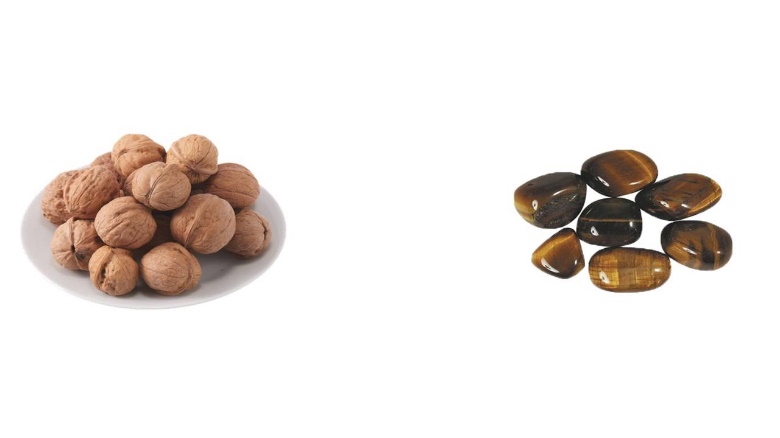  Walnuts - Stones |
| 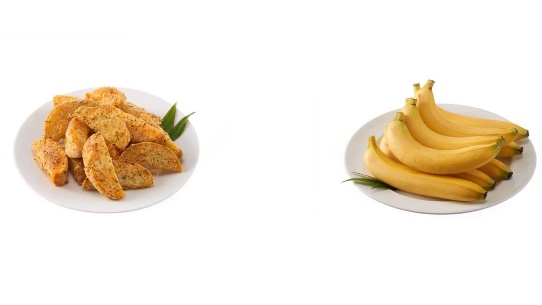  Potato wedges - Bananas | 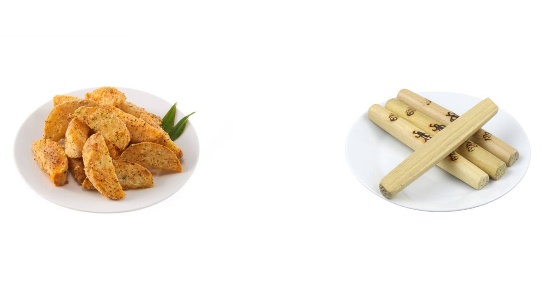  Potato wedges – Yut | 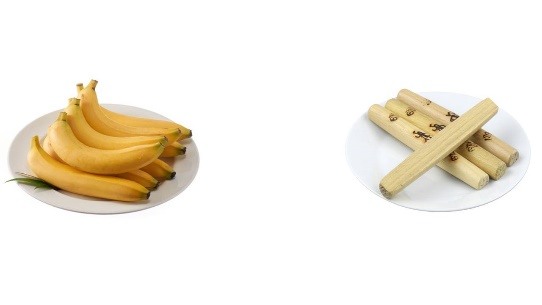  Bananas - Yut |
| 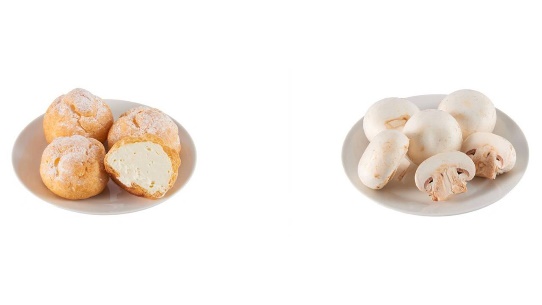  Cream puffs - Mushrooms | 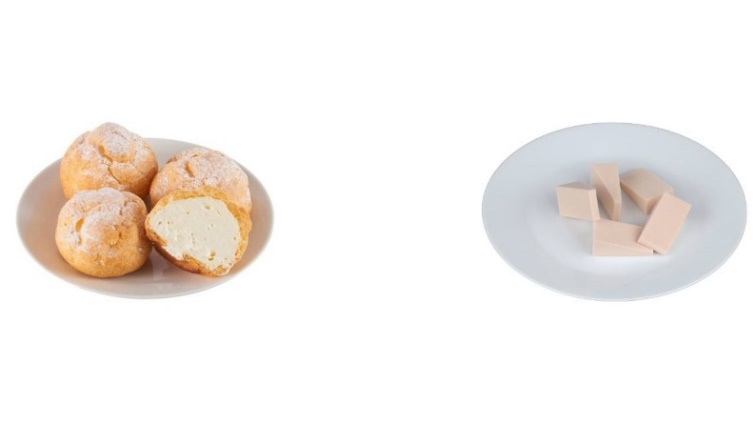  Cream puffs – Makeup sponges | 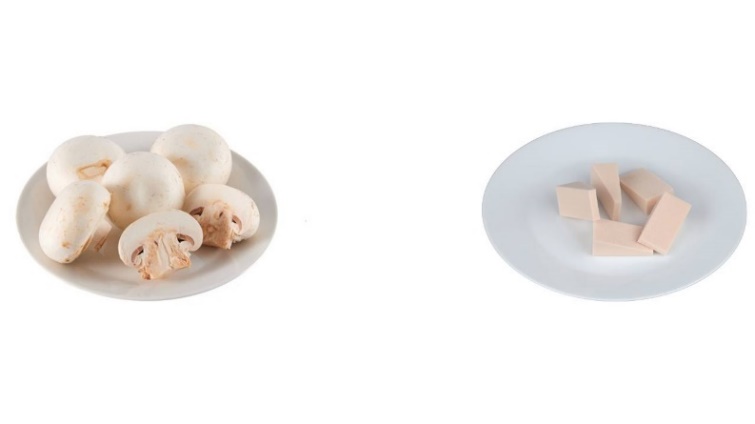  Mushrooms – Makeup sponges |
| 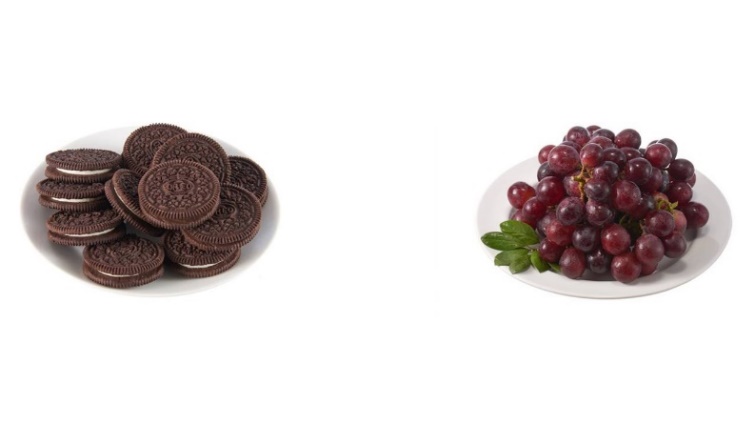  Cookies - Grapes | 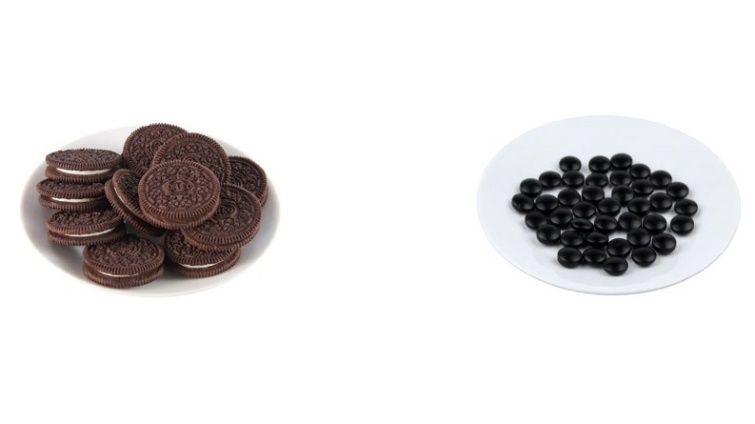  Cookies – Baduk stone | 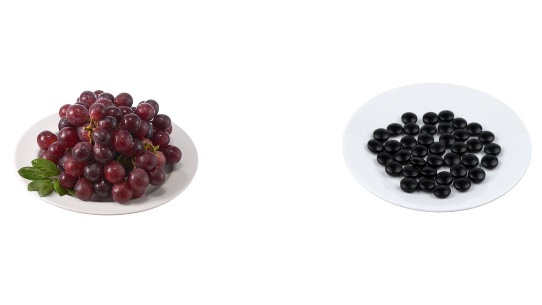  Grapes – Baduk stones |
| ***Notes.*** *Each pair was presented in a counterbalanced order, and cues were presented twice on each side of the monitor. Each pair of cues was presented at a size of 80 × 100 mm with their centers 200 mm apart. The high-calorie food cues were all items containing high quantities of fat and sugar, such as fries, ice creams, and chocolates. The low-calorie food cues included various types of vegetables and fruits.* | | |
