# Supplementary material for: Attentional Bias to High-Calorie Food in Binge Eaters With High Shape/Weight Concern
Source: Front Psychiatry. 2021 Mar 4;12:606296. doi: 10.3389/fpsyt.2021.606296 (PMC7982957; doi:10.3389/fpsyt.2021.606296)
Supplement: Supplementary Material 2 — Mean initial fixation latency for each stimulus among groups. [file Table_2.DOCX]

**Supplementary 2 |** Mean initial fixation latency for each stimuli among groups (s).

| \|  \| BE \| \|  \| HC \| \|  \| \| \| --- \| --- \| --- \| --- \| --- \| --- \| --- \| --- \| \|  \| High SWC  (*n* = 25) \| Low SWC  (*n* = 25) \|  \| High SWC  (*n* = 25) \| Low SWC  (*n* = 30) \| *F* \| \| \| High-calorie \| .57 (.34) \| .95 (.74) \|  \| .79 (.45) \| .67 (.38) \| 6.68 \| * \| \| Low-calorie \| .47 (.24) \| .81 (1.46) \|  \| .46 (.46) \| .60 (.71) \| .37 \|  \| \| Neutral \| 1.49 (2.56) \| 1.32 (2.68) \|  \| .84 (.52) \| .98 (.82) \| .17 \|  \| \| ***Notes.*** *Mean (standard deviation); * p < .05 BE = binge eaters; HC = healthy controls; SWC = shape/weight concern; High-calorie = high-calorie food cues; Low-calorie = low-calorie food cues; Neutral = neutral cues; Test Statistics (F) = results of the omnibus F-test.* \| \| \| \| \| \| \| \| |
| --- | --- | --- | --- | --- | --- | --- | --- | --- | --- | --- | --- | --- | --- | --- | --- | --- | --- | --- | --- | --- | --- | --- | --- | --- | --- | --- | --- | --- | --- | --- | --- | --- | --- | --- | --- | --- | --- | --- | --- | --- | --- | --- | --- | --- | --- | --- | --- | --- |
