# Supplementary material for: Attentional Bias to High-Calorie Food in Binge Eaters With High Shape/Weight Concern
Source: Front Psychiatry. 2021 Mar 4;12:606296. doi: 10.3389/fpsyt.2021.606296 (PMC7982957; doi:10.3389/fpsyt.2021.606296)
Supplement: Supplementary Material 3 — Mean total fixation duration for each stimulus among groups. [file Table_3.DOCX]

**Supplementary 3 |** Mean total fixation duration for each stimuli among groups (s).

| \|  \| BE \| \|  \| HC \| \|  \| \| \| --- \| --- \| --- \| --- \| --- \| --- \| --- \| --- \| \|  \| High SWC  (*n* = 25) \| Low SWC  (*n* = 25) \|  \| High SWC  (*n* = 25) \| Low SWC  (*n* = 30) \| *F* \| \| \| High-calorie \| 79.68 (16.34) \| 68.96 (19.06) \|  \| 69.94 (18.04) \| 75.30 (14.98) \| 5.79 \| * \| \| Low-calorie \| 62.35 (9.52) \| 62.80 (13.16) \|  \| 62.23 (12.41) \| 61.92 (9.62) \| .03 \|  \| \| Neutral \| 37.64 (15.49) \| 38.49 (12.72) \|  \| 36.98 (12.72) \| 37.47 (11.75) \| .00 \|  \| \| ***Notes.*** *Mean (standard deviation); * p < .05 BE = binge eaters; HC = healthy controls; SWC = shape/weight concern; High-calorie = high-calorie food cues; Low-calorie = low-calorie food cues; Neutral = neutral cues; Test Statistics (F) = results of the omnibus F-test.* \| \| \| \| \| \| \| \| |
| --- | --- | --- | --- | --- | --- | --- | --- | --- | --- | --- | --- | --- | --- | --- | --- | --- | --- | --- | --- | --- | --- | --- | --- | --- | --- | --- | --- | --- | --- | --- | --- | --- | --- | --- | --- | --- | --- | --- | --- | --- | --- | --- | --- | --- | --- | --- | --- | --- |
